# Collecting household water usage data: telephone questionnaire or diary?

**DOI:** 10.1186/1471-2288-9-72

**Published:** 2009-11-09

**Authors:** Joanne E O'Toole, Martha I Sinclair, Karin Leder

**Affiliations:** 1 Department of Epidemiology and Preventive Medicine, Monash University, The Alfred, Melbourne, Victoria 3004, Australia

## Abstract

**Background:**

Quantitative Microbial Risk Assessment (QMRA), a modelling approach, is used to assess health risks. Inputs into the QMRA process include data that characterise the intensity, frequency and duration of exposure to risk(s). Data gaps for water exposure assessment include the duration and frequency of urban non-potable (non-drinking) water use. The primary objective of this study was to compare household water usage results obtained using two data collection tools, a computer assisted telephone interview (CATI) and a 7-day water activity diary, in order to assess the effect of different methodological survey approaches on derived exposure estimates. Costs and logistical aspects of each data collection tool were also examined.

**Methods:**

A total of 232 households in an Australian dual reticulation scheme (where households are supplied with two grades of water through separate pipe networks) were surveyed about their water usage using both a CATI and a 7-day diary. Householders were questioned about their use of recycled water for toilet flushing, garden watering and other outdoor activities. Householders were also questioned about their water use in the laundry. Agreement between reported CATI and diary water usage responses was assessed.

**Results:**

Results of this study showed that the level of agreement between CATI and diary responses was greater for more frequent water-related activities except toilet flushing and for those activities where standard durations or settings were employed. In addition, this study showed that the unit cost of diary administration was greater than for the CATI, excluding consideration of the initial selection and recruitment steps.

**Conclusion:**

This study showed that it is possible to successfully 'remotely' coordinate diary completion providing that adequate instructions are given and that diary recording forms are well designed. In addition, good diary return rates can be achieved using a monetary incentive and the diary format allows for collective recording, rather than an individual's estimation, of household water usage. Accordingly, there is merit in further exploring the use of diaries for collection of water usage information either in combination with a mail out for recruitment, or potentially in the future with Internet-based recruitment (as household Internet uptake increases).

## Background

A formal risk management process is increasingly being employed in the management of drinking water supplies and has been adopted in drinking water guidelines [1,2]. It has also been used in the design of Australian guidelines for water recycling for non-potable uses [3]. From a health perspective, these guidelines principally focus on microbial hazards and use a Quantitative Microbial Risk Management (QMRA) process for guideline setting. Exposure assessment, one element in QMRA, describes the conditions conducive to human exposure to the risk and typically includes a description of the intensity, frequency and duration of exposure as well as the exposure routes and the people exposed [4]. Data gaps for water exposure assessment include the duration and frequency of urban non-potable (non-drinking) water use. Good quality contemporaneous data about the duration and frequency of water-using activities (particularly in circumstances where water restrictions may be implemented due to water shortages, as is currently the case in many parts of Australia) combined with information about the total volume per exposure event are needed to obtain the total volume exposure per person per annum. This information, in combination with the number of residual micro-organisms present in the source water and the dose response of micro-organism(s), can then be used to obtain an estimate of the annual probability of infection associated with micro-organism(s) of concern for designated water-using scenarios. Knowing the exposure volume per person per annum for a particular water-using scenario (e.g. toilet flushing) and water type, it is possible to determine the minimum level of water treatment necessary to achieve a predetermined health target and/or to determine whether substitution of one water type with another will lead to an unacceptable increase in the magnitude of risk.

The need for exposure information leads to questions about how such data are best collected. Each survey method has advantages and disadvantages associated with its use. The rationale for use of a particular survey method is often based on its practicality, cost and the complexity of the questions to be answered. This study was undertaken as a sub-set of a larger project that collected information about the duration and frequency of household recycled and drinking water use. The objective of this study was to compare household water usage results obtained using two data collection tools, a computer assisted telephone interview (CATI) and a 7-day water activity diary, in order to assess the effect of different methodological survey approaches on results. This information is important when considering the conduct of future studies, the interpretation of results of any household water usage survey and ultimately, in determining the likely precision of derived exposure estimates. Only 'direct' exposure of householders to recycled water was assessed, with no consideration given to incidental simultaneous exposure of others. Costs and logistical aspects of each survey method were also examined.

## Methods

Data were collected from households in the Rouse Hill dual reticulation scheme in Sydney Australia. The Rouse Hill scheme is the largest (approximately 16,000 households) and longest established (since 2001) dual reticulation scheme in Australia. In such schemes households are supplied with two grades of water through separate pipe networks. One grade of water is of high quality and used for drinking, cooking and other household purposes, while the other is of lower quality and used for non-potable purposes such as toilet flushing and garden watering. Whilst water restrictions were applicable to the drinking water supply during the survey period, there was no restriction on the amount of recycled water used by Rouse Hill households. Householders were questioned about their use of recycled water for toilet flushing, garden watering and other outdoor activities. In addition, householders were questioned about their water use in the laundry (currently supplied with drinking water but also a use for which recycled water may be substituted in future). An extensive survey of water-using behaviours had not been administered to Rouse Hill dual reticulation residents before this survey. The study was conducted as a University survey over the period February to April 2006 (inclusive) and was approved by the Monash University Standing Committee on Ethics and Research involving Humans (SCERH Project number 2005/659).

### Household recruitment

Australian Electoral Commission (AEC) and Electronic White Pages (EWP) records were used to select eligible dual reticulation households. In Australia, voting is compulsory and the electoral roll provides an easily accessible and up to date means of contacting persons for health research studies. Records were grouped according to electorate, suburb, postcode, street number and address, resulting in a list of households (single dwellings) located in the area of interest (defined by two postcode zones). A random subset of 3,500 households was selected from the 14,000 available. Data matching with the Electronic White Pages (EWP) was then performed for the purpose of obtaining telephone numbers. The generated random sequence of households was the order in which data matching with the EWP was performed and introductory letters were sent. Telephone matching of records for a total number of 3,500 households only was attempted as a pilot study results indicated that this would be sufficient to achieve a minimum total sample size of 500 CATI responses with 200 of these households also completing and returning a water-activity diary. These sample sizes were selected based on budgetary considerations and to allow a comparison of diary and CATI responses corresponding to a 95% confidence interval of 0.5 - 0.7 where the weighted kappa statistic is 0.6 [5].

Elector households with a listed telephone number in the EWP were sent an introductory letter inviting them to participate in the study. Telephone contact was commenced one to three weeks after the introductory letter was mailed. Four telephone contact attempts for each household were made before attempts were terminated. The majority of telephone calls were made between 6 pm and 9 pm. Once telephone contact was made, householders were invited to complete a Computer Assisted Telephone Interview (CATI). Where this was declined this was the final contact with householders. Households completing the CATI were invited to receive and complete a water-activity diary. Contact was terminated at this stage for those declining to complete a water activity diary. Those agreeing to complete a water-activity diary were sent a set of diary cards and diary instructions and were advised that a gift voucher to the value of A$40 would be posted to them following diary completion and return of the cards.

### Computer Assisted Telephone Interview

The CATI questions used for the survey [see additional file 1] were based on a previous Australian household water activity survey [6].

As recycled water in Rouse Hill dual reticulation households is used for toilet flushing and outdoor water use, the CATI was designed to include questions about these activities. Whilst recycled water is not plumbed into Rouse Hill dual reticulation households for machine washing (drinking water is used for this purpose), the CATI was also designed to include questions relating to laundry activities as a scoping exercise to explore the use of recycled water, rather than drinking water, for laundry purposes. The questions posed to householders about the duration of the water-using activities required them to estimate the duration in minutes. For questions about the frequency of a water-using activity, householders were required to classify the frequency of their water usage into pre-defined categories, or in some instances (e.g. toilet flushing, laundry loads and garden watering) to give a number estimate for the 7-days immediately prior to the interview. Since the 1980's, as a water saving measure, Australian regulations have mandated that dual flush toilets are installed in new homes and when existing homes are renovated. Accordingly, respondents were asked to give a number estimate for both half and full toilet flushes per day. Respondents were also required to indicate the machine washing settings (water level, water temperature) commonly used in the household. A total of 523 households completed the CATI.

### Water activity diary

The water activity diary took the form of 'diary cards' [see additional file 2]. Five different diary cards were produced, each one relating to a particular activity as follows: Household characteristics, toilet use, garden watering, outside water use (excluding garden watering) and laundry use. Each card provided for 7-days recording. The recording of activities required one or more of the following:

• A tick to indicate the frequency of use (e.g. number of toilet flushes (half and full flushes), number of machine washing loads, washing machine settings)

• Circling an option (e.g. washing machine type)

• Entry of a number (e.g. the number of minutes that an activity had been performed)

• The entry of a code (e.g. specification of WCB for watering can or bucket in the outdoor usage diary card with codes specified at the bottom of the card)

• Free-hand written specification of the type of water activity (e.g. for the outdoor usage card where the activity was not already specified on the card) or location of toilet (e.g. ensuite bathroom)

A diary instruction pamphlet was prepared and for clarity of instructions, diary cards were identified by both type and colour. In addition, examples of diary entries for each card were pre-printed on the reverse of each diary card. The diary instructions referred to these examples and provided further explanation. To facilitate the recording of activities as they occurred, it was suggested that the diary cards be placed at convenient locations in the house that made them accessible for recording purposes. For example, it was suggested that the toilet card might be placed on each toilet door. Water proof covers, pens with which to complete diaries and adhesive were provided in each diary pack to support diary completion. In addition, a reply-paid envelope was included with the diary pack to support return of completed diaries. A follow up call was made to householders a few days after mailing the diary pack to check that it had been received, and householders were requested to complete and return the diaries within 4 weeks. There was no further contact with households during the diary recording period.

### Data analysis

CATI responses were entered into an ACCESS (Microsoft Office 2000) database at the time of the telephone interview. Information from each of the diary cards was entered onto an ACCESS (Microsoft Office 2000) diary database which was constructed so that the appearance of the diary cards and the database entry screens were similar. The entry of data from toilet flushing and laundry cards required some minor processing. For the toilet and laundry card this consisted of summing the number of ticks on each of the cards to arrive at tallies for each activity per week. At least 10% and up to 25% of data entry, depending on card type, was checked by a second operator.

Diary numerical entries of the frequency of a water-using activity were classified into the same categories as used for the CATI where there was discordance in data type between the CATI (categorical) and diary (numerical, continuous). For toilet flushing, the number of half and full toilet flushes per household were tallied from diary cards to give a total number of half and full flushes per household per week. For the CATI the number of full and half flushes per day, as estimated by the respondent, was converted to total number of full and half flushes per household per week assuming the same daily toilet flushing estimate for all household members.

Agreement between reported water usage using the CATI and diary responses was measured using a weighted kappa statistic, which considers disagreement close to the diagonal less heavily than disagreement further from the diagonal. Weighted kappa results were classified as 0.00 - 0.40 = 'poor'; 0.41 - 0.75 = 'Fair to good' and 0.76 - 1.00 = 'Excellent' [7]. Bland-Altman scatter plots were used to visually compare the measures of water usage [8]. Statistical analysis was carried out using STATA version 9 (STATA™ Stata Corporation, Texas, USA).

## Results

### Recruitment

Of the 523 dual reticulation eligible households completing a CATI, 371 (70%) agreed to complete a water activity diary. All 371 households agreeing to complete a diary were sent water activity diary cards and 232 (63%) households returned completed cards within a 4 week period and were eligible for the A$40 voucher offered as an incentive.

### Timing of the CATI and diaries

Administration of CATIs and diaries with the exception of two CATI and seven diaries was compacted into two overlapping periods each of seven weeks duration. There was a two week lag between the administration of the first batch of CATIs and commencement of the first batch of diaries. All diaries except one were commenced within 3 weeks of the cessation of the telephone interviews. The total survey period was 12 weeks.

### Comparison of CATI and diary characteristics and time input

A summary of CATI and diary characteristics, costs, data handling procedures and estimated number of hours for completion of each are given in Table 1.

**Table 1:** Summary of CATI and diary characteristics and data handling prior to statistical analysis

Characteristic	CATI	Diary
Database design A$	$5000	$2360

Duration	15-20 min*	Not applicable

Target number	500	200

Order of administration	First	Second (always Post CATI)

Recruitment strategy	Electoral records and introductory letter	$40 incentive (voucher)

Time period under investigation	Immediate past week (in detail) and up to one year prior	7-day period only

Data entry onto ACCESS database	Immediate (as telephone interview was conducted)	Manual entry

Actions prior to interview/diary administration excluding initial mail-out and associated activities	- Questionnaire design	- Print diary cards
	- ACCESS database design	- Design and print diary instructions
		- Compile diary cards; pack and send*
		- Follow-up phone call to check diary pack receipt*
		- ACCESS database design

Actions subsequent to interview/diary completion	- Check data entry immediately following interview*	- Check diary cards for completion/legibility*
		- Tally number of uses recorded on cards*
		- Transfer data including tallies from diary card to ACCESS database*
		- Check 10-25% data transferral to database*

Estimated number of hours labour per completed CATI/diary (for * activities)	30 min	1 hr 25 min

### Outdoor water use

**Figure 1:**
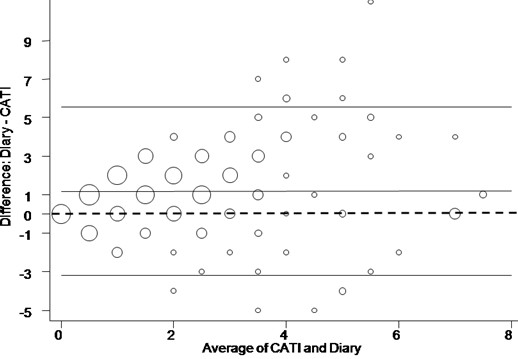
**Number of times garden watered in past 7 days**. Scatter plot (Bland-Altman plot) of diary response minus CATI response (vertical axis) against average of CATI response and diary response (horizontal axis). The centre full horizontal line corresponds to the average difference and upper and lower full horizontal lines correspond to the 95% limits of agreement. The dashed straight line represents the line of zero difference. The larger the data points (circles) the higher the number of households that recorded these values.

Table 2 gives a summary of CATI and diary responses for the garden irrigation module. These results show lower CATI results for: number of garden watering sessions in prior seven days, duration of automatic water system use and duration of hand held hose use. Higher CATI results were obtained for: duration of use of fixed manual systems and hose and sprinkler watering sessions. A comparison of CATI and diary responses showed poor agreement between data collection tools for all but the duration of the automatic watering system, which showed 'fair to good' agreement (weighted kappa = 0.51). Agreement between data collection tools for the total number of garden watering sessions in the 7-days prior, as measured by a weighted kappa (0.36), was 'poor'. The Bland-Altman scatter plot (Figure 1) for garden watering frequency in each of the 7-day CATI and diary survey periods shows that the variance increased in proportion to the average values. The average difference between the Diary and CATI responses (the diary was higher) was 1.18 (Confidence interval 0.89 to 1.47).

**Table 2:** CATI versus Diary responses: Garden irrigation

Question	CATI response N = 219		Diary response N = 219		CATI response relative to Diary weighted kappa (agreement*)
	Average (standard deviation)	% using (N)	Average (standard deviation)	% using (N)	
Number of garden watering sessions last 7 days	1.7 (1.79)	72.2% (158)	2.9 (2.24)	86.3% (189)	0.36 (poor)
Automatic watering system duration use (min)	38.0 (19.2)	15.5% (34)	55.4 (26.4)	11.9% (26)	0.51 (fair to good)
Hand held hose duration use (min)	26.3 (16.9)	40.6% (89)	26.5 (32.7)	58.0% (127)	0.36 (poor)
Fixed manual system duration use (min)	45.3 (23.9)	17.4% (38)	43.2 (17.12)	12.8% (28)	0.21 (poor)
Hose and sprinkler duration use (min)	60.2 (24.8)	13.2% (29)	54.6 (33.5)	22.8% (50)	0.05 (poor)

* Fleiss JL: Statistical methods for rates and proportions 2^nd ^edition, New York; Wiley; 1981:218

### Laundry use

**Figure 2:**
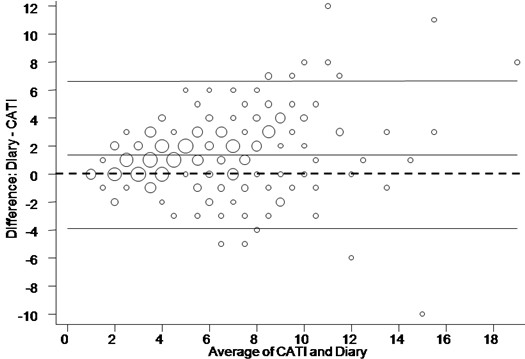
**Number of washing machine loads per household per week**. Scatter plot (Bland-Altman plot) of diary response minus CATI response (vertical axis) against average of CATI response and diary response (horizontal axis). The centre full horizontal line corresponds to the average difference and upper and lower full horizontal lines correspond to the 95% limits of agreement. The dashed straight line represents the line of zero difference. The larger the data points (circles) the higher the number of households that recorded these values.

Agreement between the CATI and diary responses for the number of washing machine loads per week was 'fair to good' as measured by a weighted kappa of 0.66 (Table 3). The Bland-Altman scatter plot (Figure 2) for number of laundry loads per week is symmetric for all but low average values. The average difference (the diary was higher) in the number of machine washing loads per week between the diary and CATI was 1.37 (Confidence interval 1.02 to 1.73). Statistical analysis of the agreement between responses relating to washing machine water level showed 'poor' agreement between data collection tools relating to the selection of low (weighted kappa = 0.24) and medium (weighted kappa = 0.33) water levels and 'fair to good' agreement for high (weighted kappa = 0.44) and automatic (weighted kappa = 0.64) water level settings. Statistical analysis of agreement between CATI responses and the diary relating to water temperature selection showed 'fair to good' agreement relating to selection of cold wash (weighted kappa = 0.62) and warm wash (weighted kappa = 0.58) but only 'poor' agreement for hot wash (weighted kappa = 0.17).

**Table 3:** CATI versus Diary responses: Laundry

Statistic	Number of washing machine loads per week		CATI response relative
	CATI response (Median 95% confidence limit)	Diary response (Median 95% confidence limit)	to Diary weighted kappa (agreement*)
**Average**	5.3	6.7	
**Standard deviation**	3.1	3.7	
**Median**	5 (4 - 5)	6 (5.5 - 7)	0.66 (fair to good)
**N**	217	217	

* Fleiss JL: Statistical methods for rates and proportions 2^nd ^edition, New York; Wiley; 1981:218

### Toilet flushing

**Figure 3:**
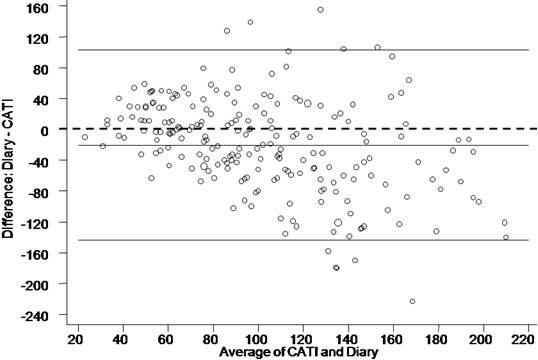
**Toilet flushes per household per week**. Scatter plot (Bland-Altman plot) of diary response minus CATI response (vertical axis) against average of CATI response and diary response (horizontal axis). The centre full horizontal line corresponds to the average difference and upper and lower full horizontal lines correspond to the 95% limits of agreement. The dashed straight line represents the line of zero difference. The larger the data points (circles) the higher the number of households that recorded these values.

Agreement between data collection tools for the total number of toilet flushes per household per week, as measured by a weighted kappa (0.29), was 'poor' (Table 4). The Bland-Altman scatter plot (Figure 3) shows that there was a tendency for greater (negative) differences (diary response lower than the CATI response) with greater average number of toilet flushes per household per week. For the total number of toilet flushes a mean difference between the Diary and CATI estimates of -20.6 (Confidence interval -28.9 to -12.3) was recorded. Table 5 presents estimates of the frequency of use of half flush as compared with full flush by the CATI and diary. A weighted kappa statistic of 0.36 (poor) was obtained when CATI and diary responses were compared for half flush frequency estimates.

**Table 4:** CATI versus Diary responses: Toilet flushing

Statistic	Total number of toilet flushes per household per week		CATI response relative to Diary
	CATI response (Median 95% confidence limit)	Diary response (Median 95% confidence limit)	weighted kappa (agreement*)
**Average**	112.4	91.8	
**Standard deviation**	60.5	40.1	
**Median**	105 (96 - 112)	85 (77 - 90)	0.29 (poor)
**N**	216	216	

* Fleiss JL: Statistical methods for rates and proportions 2nd edition, New York; Wiley; 1981:218

**Table 5:** CATI versus Diary responses: Toilet flushing

Diary	CATI: Proportion of time personally use half flush				
Proportion of time use half flush	= <25%	25-50%	51-75%	= >75%	Total
**= <25%**	5	2	2	3	12
**25-50%**	5	0	6	9	20
**51-75%**	7	13	25	65	110
**= > 75%**	0	4	14	61	79
**Total**	17	19	47	138	221

## Discussion

When using QMRA techniques to aid in the development of health-based quality targets for waterborne pathogens it is important that input data have validity. One of the inputs required for QMRA is accurate exposure information, which raises questions about how this information is best collected. Validity of a survey method can be quantified by comparing survey responses obtained with a gold standard. For example, in health-related studies where exposure to a particular agent is being assessed, responses to questions about exposures may be compared with results of tests for relevant biological markers. For studies such as this one where water used by householders inside and outside the home for non-potable purposes was the subject of investigation, the true gold standard is the volume of water householders are exposed to during specific water-using activities. Whilst sophisticated water meters are available that can, in theory, measure the volume of in-house and outside house water usage at individual taps, their use is somewhat problematic in an extensive household water usage survey. This is because of cost and logistical considerations associated with meter installation to a large number of households, meter calibration (e.g. to match water usage events such as toilet flushing or showering with recorded data 'spikes') and the lack of sensitivity of meters to detect single tap usage [9]. In addition, the volume of water used at a particular tap is not a true measure of exposure because factors such as human behaviour and the type of water-using equipment also determine the exposure volume.

As a consequence of the barriers associated with accurately measuring individual domestic water exposure, this study sought to collect water usage information using two alternative data collection tools, a telephone interview and water-activity diary, both previously used for Australian household surveys [6,9,10] and compare results. In this study, the comparison of these data collection tools was based not only on estimates of the duration and frequency of water-using activities but also on the cost and logistics of administration of each survey type.

### Exposure estimates

The highest level of agreement between CATI and diary responses was obtained for: number of washing machine loads per week (Table 3); automatic system watering session duration (Table 2); use of high and automatic washing machine levels and use of cold and warm washing machine wash temperatures. Lower levels of agreement between CATI and 7-day diary results were obtained for less frequently performed water-using activities with the exception of toilet flushing (frequently performed but also showing poor agreement) or where standard durations (e.g. as might occur with automatic pre - programmed systems) or frequently applied settings were not used. 'Poor' agreement was obtained for: number of garden watering sessions; hand held hose watering duration; fixed manual water system duration; hose and sprinkler watering duration (Table 2); washing machine hot water wash; washing machine low water level and washing machine medium water level. Overall, when considering all individual water uses and the relative performance of the diary and CATI, diary responses were not consistently higher or lower than CATI responses.

The poor agreement between diary and CATI responses for the total number of toilet flushes per household per week (weighted kappa = 0.29) (Table 4) and for the frequency of use of half flush as a percentage of total toilet flushes (weighted kappa = 0.36) (Table 5) may be attributed to a number of factors. Considering that the number of toilet flushes is not insignificant per week per individual, it is likely that the exact number at home is difficult to recall. In addition, the CATI respondent was asked about individual behaviour, yet the diary recorded household behaviour. Accordingly, the number of toilet flushes per week estimated by the CATI respondent was multiplied by the number of persons in the households and compared with the tallied entries for toilet flushing recorded in the diary. A comparison of diary and CATI results (Figure 3) showed differential bias namely, that the CATI estimate (single respondent) combined with an assumption of identical toilet flushing behaviour by all persons in the household, overestimates the total flushes in large households.

The higher estimates of the frequency of use of half flush as a proportion of all toilet flushing for the CATI as compared with the diary (Table 5) indicates that the CATI respondent believed that the use of half flush was more prevalent in the household than was the case for the 7-day diary recording period. The CATI responses may represent the desired target behaviour of the respondent rather than actual household practice or, may truly reflect the respondent's behaviour, which is different to the rest of the household.

Many of the activities showing 'poor' agreement between data collection tools, as measured by the weighted kappa statistic, were not only less frequently performed activities, but also outdoor water-related activities. A possible reason for poor agreement between results for outdoor activities is that climatic conditions may have been different for each of the CATI and diary 7-day survey periods. Whilst the majority of diaries and CATIs administered were each compacted into separate seven week periods with no greater than a 3-week lag period between cessation of telephone interviews and commencement of diaries, the climatic conditions during diary and CATI survey periods may have been significantly different for a proportion of households (e.g. rain during the CATI 7-day survey period and no rain during the diary 7-day survey period or vice-versa).

The observation of greater concordance between CATI and diary responses for more frequent and standard water-related activities except toilet flushing may reflect the greater likelihood that standard frequencies, durations and settings are known to all adult household members. This observation is supported by results from other water-related studies where completion of a questionnaire also preceded the diary and referenced a different 7-day period [11,12]. These studies attributed lower correlations between questionnaire and diary responses to a greater potential for daily variation in some water-related activities compared with established routine ones.

When all results of this study are considered, they show that different estimates of the duration and frequency of water-related activities are obtained depending upon the survey method used. For estimates of garden watering frequency and the number of laundry machine loads per week, the diary, compared with the CATI, showed general positive bias (i.e. the diary response was consistently higher). Such differences between responses for CATI and diary may be associated with one or more factors including: recall bias of the CATI respondent [13]; natural variation in water-related activity that occurs from week to week (CATI and diary responses 'referenced' different 7-day periods); the survey period may not have been over a sufficient period to 'capture' the exposure of interest (e.g. if garden watering was performed every 1.5 weeks it may not have been included in either (or both) of the diary or CATI survey periods); computations to convert single respondent results to household results (not all household members behave in the same way) and failure to complete diaries prospectively as intended or to record all activities and events [14].

Compared with a telephone interview, the use of a household diary has a clear advantage in determining household exposure to a particular water supply. This is because the diary provides a collective measure of household exposure compared with an individual's estimate of household exposure. Acknowledging that the household diary cards may have in some instances been completed by one individual, it is more likely that they were completed by the householder performing the activity(ies) of interest. This assumption is based on the cards being placed in the location of water use (if instructions were followed) and/or that responsibility for diary card recording is likely to have been allocated within the household, to the household member with greatest familiarity with household water-using practices. In contrast, the telephone questionnaire may have been answered by an adult not fully familiar with the household water usage.

The prospective recording of water-related activities using a diary is also another advantage of using a diary collection method compared with a CATI. Diary responses are not subject to recall bias (assuming prospective completion) hence it is probable that the diary information provides the more accurate figure, compared with the CATI. Recall bias may be responsible for either under or overestimation as respondents may forget relevant episodes or they may report an episode from outside the period of interest as if it had happened within the period (forward telescoping) or vice versa (backward telescoping) [13]. In this study, the CATI gave the higher figure for toilet flushing (this figure was also influenced by conversion from an individual estimate to a household one) and lower figures for garden watering frequency and number of machine washing loads. In addition, the diary format has the advantage that it provides continuous numerical, rather than categorical, data for use in QMRA modelling. In contrast, the CATI for some water-using activities provided only categorical data.

### Logistics and Cost

Given advantages conferred by use of a diary, rather than telephone interview for obtaining household exposure information, this leads to questions about how to overcome obstacles such as cost and logistics, commonly associated with diary administration. In this study, the estimated number of input researcher hours per completed diary was 2-3 times greater than that for each completed CATI (Table 1). The time estimate for the completion of each was up until statistical analysis of data was performed but was not inclusive of the initial recruitment process which was common to both the CATI and the diary; diary recruitment being dependent upon CATI uptake. The researcher time input per CATI was approximately 30 minutes, based on the interview time (15-20 min), look-up of household details and telephone contact information prior to the telephone call and verification of data (data was checked immediately after the interview).

The CATI time estimate assumes that one telephone call will result in a completed CATI. In fact this was not the case as some households required more than one call to be made before a CATI (44%) was generated (or refused) and a percentage of calls did not yield a CATI (61%)[15]. However, the assumption that one telephone call yields one CATI allows a more equitable comparison with the researcher time input for data preparation and handling following diary completion. This is because the starting point for the diary administration was agreement by the CATI respondent (70% agreed) that they wished to complete a water activity diary. Thus, each diary pack sent was expected to yield a completed diary that was returned to researchers. In fact this was not the case as the return rate (within the required 4 week time frame) of diaries was 63%. Based on this return rate and using a target of 200 completed diaries, the researcher time estimate per completed diary is approximately 85 minutes. This time estimate (expressed per unit diary) is based on: compilation of 320 diary packs, follow-up phone calls to 320 households to verify diary receipt; checking of completeness and legibility of 200 completed diary packs following receipt, tallying of data prior to entry onto data base, manual transfer of data to databases and data checking and verification.

A notable difference between the CATI and diary was that an incentive payment was offered for completion of the diary but not for completion of the CATI, adding to the budget costs for the diary. The use of a monetary incentive has been successful in other Australian studies [10] and was considered appropriate to compensate householders for recording their water usage over a 7-day recording period (a significant time commitment for the respondent) but not for a 15-20 minute CATI.

In this study, manual entry of data was performed, adding to the total researcher time input required for data entry to the diary database. However, it is possible that this time input could have been reduced if data scanning of diary records had been employed, reducing both the time requirement for data transfers into the ACCESS database and for data checking. Clearly a change such as this would require custom design of data input sheets and the corresponding data base. Whilst this would add to the cost of diary data base design, it is likely that it would not exceed that of the CATI database design. However, even if data scanning was implemented, the overall cost of diary administration would nonetheless exceed that of a CATI based on the cost of incentive payments and remaining labour costs associated with the preparation, sending and sorting of diary recording sheets.

A notable observation of this study was the quality of diary card completion, indicating that the diary cards were well designed (based on cards used in a prior Australian study [6]) and were easy to complete. The fact that there was no contact with households sent diary cards except for an initial phone call confirming diary card receipt and reinforcing that diary cards should be completed within a four week period was also testament to the clarity of diary instructions. This illustrates that it is possible to 'remotely' coordinate diary completion providing that adequate instructions are given and that diary recording forms are well designed. These observations are not only relevant to diary cards mailed to participants, but by extrapolation are also pertinent to web-based diary completion.

### Limitations

This study was subject to a number of limitations including the sequential recruitment strategy in which householders were firstly recruited for the CATI and then (once the CATI was completed) were recruited to complete a 7-day water activity diary. This meant that the CATI 7-day period, always preceded the 7-day recording period for the diary. This limitation was countered as best as possible by confining the survey period to a maximum of 12 weeks (the summer period in which maximum household water consumption occurs). In addition, to reduce the time period between CATI and diary completion, a monetary incentive was offered to those households returning a completed diary within a 4 week period. Despite these measures, there was an approximate two to three week lag between CATI completion and diary commencement. The implications of this lag period are considered to be minimal for indoor water uses, which generally remain constant irrespective of weather conditions. However, this lag period potentially impacts on outdoor water uses such as garden watering where rainfall events and variations in temperature can influence garden irrigation frequency and duration. Rainfall in each of the CATI and diary 7-day periods was not tracked for individual households; hence the role of weather conditions on poor agreement between data collection tools for garden watering frequency was not able to be elucidated.

Another consequence of sequential recruitment was that only a subset of the household population that completed CATIs also completed diaries. It is therefore possible that the household population completing both the CATI and diary may have differed in some way from those that completed the CATI only. It is notable however that there was a high preparedness for householders to complete the diary (70% agreed) and that 63% of those agreeing to complete the diary also returned the diary within 4 weeks (a higher return rate may have resulted had the diary return been extended). The high rates of agreement to complete the diary support the view that financial reward was not a defining factor separating households that completed the diary from those that did not. Furthermore, the recycled water area in which householders were located is a niche housing development located in an area of high socio-economic status and is relatively homogenous with respect to household demographics and income.

Whilst it can be argued that it is likely that the diary population is representative of the larger household population completing the CATI only, a further question is whether the population completing the CATI are representative of the whole population of Rouse Hill dual reticulation households, including those that did not complete the CATI. In this study, CATI response rates were dependent upon the rate of matching of EWP and AEC records. The limiting factor in relation to household coverage was that only 57% of households were listed in the EWP, despite AEC records providing 87% of household coverage [15]. While it is possible that the water-using behaviour of listed and non-listed households in the EWP does vary, we consider that EWP listing is unlikely to be a primary determinant in the volume of water used by households. The relative uniformity in Rouse Hill dual household characteristics that drive water usage such as garden area, garden age and household size in the entire target survey area independent of EWP listing supports this assumption. Nonetheless, the possibility that the subset of households completing the diary and/or CATI were different from other households, based on a greater interest in water sustainability and advocates of higher recycled water use (and thus with a greater preparedness to devote time to diary and/or CATI completion), cannot be discounted.

Another limitation of this study was that there was no independent measure of the frequency and duration of individual water-using activities; hence the validity of both data collection tools is uncertain. It is therefore possible that both questioning via the CATI and recording of water usage using the diary were potentially subject to respondents reporting aspirational, rather than actual, behaviour. This is somewhat unlikely as this study was undertaken independently of the water authority supplying recycled and drinking water to householders and there was no disincentive for householders to report their actual water behaviour. Also, even though there were water restrictions relating to the use of drinking water outside the home during the study period they did not apply to inside use or to recycled water use; encompassing those water uses under investigation in this study.

Even though there were no adverse ramifications for the householder associated with reporting actual water usage, it is possible that drinking water restrictions may have influenced recycled water use despite lack of restrictions for recycled water. Such behavioural modifications are not predictable and are best addressed by the collection of contemporaneous water usage data. In doing this, the impact on exposure profiles, of behavioural changes associated with changing community attitudes and newly introduced water-using appliances, can be assessed.

## Conclusion

The collection of water usage data is important to address data gaps for the assessment of the distribution of exposure of the human population in specific localities to contaminants in water through non-potable water use. This study showed that it is possible to successfully 'remotely' coordinate diary completion providing that adequate instructions are given and that diary recording forms are well designed. In addition, good diary return rates can be achieved using a monetary incentive and the diary format allows for collective recording, rather than an individual's estimation, of household water usage. Given reduced response rates associated with telephone databases, possible future alternative methods for data collection using diaries which by-passes telephone databases is to approach potential households by mail or via the Internet (as household Internet access increases). When exploring these options, particular attention should be paid to response rates in addition to diary costs, which in this study were greater than for the CATI.

## Competing interests

The authors declare that they have no competing interests.

## Authors' contributions

JO was responsible for the administration of both the diary and telephone surveys, design of the paper and analysis and interpretation of data. MS and KL were involved in the design concept of the project, drafting and revision of the final manuscript for intellectual content. All authors had full access to data in the study and had final responsibility for the decision to submit for publication. All authors have read and approved the final manuscript.

## Pre-publication history

The pre-publication history for this paper can be accessed here:

http://www.biomedcentral.com/1471-2288/9/72/prepub

## Supplementary Material

Additional file 1 **Computer Assisted Telephone Interview (CATI) questions**. This file contains the CATI preamble script and questions posed to householders about their water usage. Click here for file

Additional file 2 **Diary cards**. This file contains the diary cards (five different cards) sent to householders that comprised the water-activity diary. Click here for file
