# Supplementary material for: Collecting household water usage data: telephone questionnaire or diary?
Source: BMC Med Res Methodol. 2009 Nov 9;9:72. doi: 10.1186/1471-2288-9-72 (PMC2777918; doi:10.1186/1471-2288-9-72)
Supplement: Additional file 1 — Computer Assisted Telephone Interview (CATI) questions. This file contains the CATI preamble script and questions posed to householders about their water usage. [file 1471-2288-9-72-S1.pdf]

Appendix 1: Computer Assisted Telephone Interview – Preamble script and Questions

Preamble script at start of CATI (on screen)

Hello, my name is ……………………and I am ringing from the Monash University medical school (in Victoria) about a recycled water project that we are conducting.

We are performing research into the use of recycled water in homes as a conservation measure and its potential health effects.

In the last month we sent a pamphlet to your address explaining our study and wonder whether you could assist us by answering a 20 minute questionnaire on your household water usage such as washing machine and toilet use and garden watering.

If Yes and can do interview now ask eligibility questions: Ask recycled water (dual reticulation households) both eligibility questions. Control households only to be asked about their age ie >18 years old.

Firstly, I have to confirm that you are eligible for the telephone interview:

Only recycled water (dual reticulation households) to be asked this eligibility question

- Is this household supplied with recycled water

If Yes = eligible

If No = in eligible
If ineligible thank person for their help

Ask both recycled (dual reticulation) and control households this eligibility question:

- Are you >18 years old?

If Yes = eligible
If No = ask to speak to householder >18 years old

If Yes but can't do interview now:
Arrange to call back at a convenient time (ascertain name of person to be contacted and time that will suit)
Person to ask for:…………………………………………………..

Most convenient time to call back:………………………………………

If No:
Thank the householder and say goodbye

If eligible:
You are eligible for the telephone interview so now I will begin the interview:

Computer Assisted Telephone Interview –Questions

GENERAL QUESTIONS MODULE	
**1. How many people 18 years or older live in the household?**	#
**2. Are there any children under 18 years old?**<br>▪ Yes **(Go to a)**<br>▪ No **(Go to Question 3 this module)**	☐<br>☐
**a) How many children in each of the age ranges?**<br>▪ Under 5 years<br>▪ 5-12 years<br>▪ 13-17years	#<br>#<br>#
**3. How many persons are usually at home <u>during the day</u> on weekdays?**	#
4. **Are you the property owner or tenant**?<br>▪ Property owner<br>▪ Tenant	☐<br>☐
**5. What type of house do you live in? Is it a separate house or a unit or terrace style house ? (Interviewer to tick relevant box as below)**<br>▪ Separate house<br>▪ Semi detached house<br>▪ A town house or unit<br>▪ Row / terrace style house<br>▪ Flat or apartment<br>Other (specify)…………………………………………	☐<br>☐<br>☐<br>☐<br>☐<br>☐
**6. How many drinking water taps are there outside the home? (If control household, Go to GARDEN MODULE after answering this question)**	#
**<u>RECYCLED WATER HOUSEHOLDS ONLY</u>:**<br>**7. How many <u>recycled</u> water taps are there outside the home?**<br>**Go to GARDEN MODULE**	#

GARDEN MODULE	
**a) What is the garden size?**<br><br>• No garden (**Go to TOILET MODULE**)<br><br>▪ Occupies <10% of the land area of the property<br><br>▪ Occupies 10-25% of the land area of the property<br><br>▪ Occupies >25% of the land area of the property	☐<br>☐<br>☐<br>☐
**b) Describe the type of garden**<br>▪ Courtyard style with paving or decking<br><br>▪ Lawn only<br><br>▪ Lawn and plants<br><br>▪ Plants or vegetable garden only	☐<br>☐<br>☐<br>☐
**c) How many years has your garden been established?**<br><br>• Enter number of years<br><br>• Don't know	# ☐
**d) Do you or anyone in the household water the garden at any time?**<br><br>▪ Yes **(Go to e if dual reticulation household otherwise if in control group go to f)**<br><br>▪ No (**Go to TOILET MODULE)**	☐<br>☐
**ONLY ASK DUAL RETICULATION HOUSEHOLDS THIS QUESTION**<br><br>**e) What type of water do you use to water the garden?**<br><br>• **Recycled water only**<br><br>• **Drinking water only**<br><br>• **A mixture of recycled water and drinking water**	☐<br>☐<br>☐
**f) Are you able to answer questions / make estimations about garden watering**<br>▪ **No (Go to TOILET MODULE)**<br><br>▪ **Yes (Go to g)**	☐<br>☐

**g) Think back over the last 7 days: did you or anyone else in the household water the garden or lawn?**<br><br>• Yes **(Go to h)**<br><br>• No **(Go to GARDEN QUESTION 2)**	☐<br>☐
**h) How many times did you or someone else water the garden in the last 7 days?**<br>**(If answered 'once' then Go to i, If not Go to j)**	#
**i) If you or someone else only watered the garden once in the last 7 days how long ago before that was it last watered?**<br><br>▪ Between 1-2 weeks ago<br><br>▪ More than 2 weeks but less than 4 weeks ago<br><br>▪ More than a month ago	☐<br>☐<br>☐
**j) In which of the following ways did you or someone else in the household, water the garden or lawn in the last 7 days?**<br><br>▪ Fixed automatic watering system<br><br>▪ Fixed watering system operated manually<br><br>▪ Hand held hose<br><br>▪ Hose and sprinkler<br><br>▪ Watering can or bucket<br><br>▪ Other<br>**Specify…………………………**	Y/N<br><br>☐<br><br>☐<br><br>☐<br>☐<br>☐<br><br>☐
**k) For how many minutes (or how many watering cans or buckets) would each watering session have lasted for each method?**<br>• Fixed automatic watering system<br><br>• Fixed watering system operated manually<br><br>• Hand held hose<br><br>• Hose and sprinkler<br><br>• Watering can or bucket<br><br>• Other	#<br><br>#<br><br>#<br><br>#<br><br>#<br><br>#

**l) To the best of your knowledge, what was the maximum number of people in the garden when the garden was being watered (excluding the person watering the garden)? *(Enter number)***<br><br>**GO TO TOILET MODULE**	#
**1. If you did not water the garden or lawn in the last 7 days think about the occasion before that when you watered the garden or lawn**<br><br>**a) When was this?**<br><br>▪ Between 1-2 weeks ago<br>▪ More than 2 weeks but less than 4 weeks ago<br>▪ More than a month ago	☐<br>☐<br>☐
**b) In which of the following ways did you or someone else in the household, water the garden or lawn on this occasion:**<br><br>• Fixed automatic watering system<br>• Fixed watering system operated manually<br>• Hand held hose<br>• Hose and sprinkler<br>• Watering can or bucket<br>• Other?<br>**Specify………………………….**	Y/N<br><br>☐<br>☐<br>☐<br>☐<br>☐<br>☐
**c) For how many minutes (or number watering cans/buckets) would each watering session have lasted for each method?**<br>• Fixed automatic watering system<br>• Fixed watering system operated manually<br>• Hand held hose<br>• Hose and sprinkler<br>• Watering can or bucket<br>• Other	#<br>#<br>#<br>#<br>#<br>#

**d) To the best of your knowledge, what is the maximum number of people were in the garden when the garden was being watered (excluding the person watering the garden)?**<br><br>**GO TO TOILET MODULE**	#

TOILET MODULE	
About your toilets<br>**1. How many toilets in your household?**	#
**2. How many of these are dual flush?**	#
**3. What percentage of the time do you use half flush as opposed to full flush?**<br>• Less than 25% of the time<br>• 25-50% of the time<br>• 51-75% of the time<br>• More than 75% of the time?	☐<br>☐<br>☐<br>☐
**4. On a normal weekday how many times would you estimate that you personally flush the toilet? Would that be the same for weekends or is it more or less frequently? What is your estimate for the weekend days?**<br>**a) on the week days**<br>**b) on each day of the weekend**<br>**GO TO SWIMMING POOL MODULE**	#<br>#

SWIMMING POOL MODULE	
**1. Do you have a swimming pool, outdoor spa or a wading / toddler pool?**<br><br>• **Yes (If control household go to 2 but if you are a dual reticulation household go to 1a)**<br><br>• **No (Go to PET MODULE)**	☐<br>☐
**ONLY DUAL RETICULATION HOUSEHOLDS ARE ASKED THIS QUESTION**<br><br>**a) What water do you use to fill the pool?**<br><br>▪ Recycled water only<br><br>▪ Drinking water only<br><br>▪ A mixture of recycled water and drinking water	☐<br>☐<br>☐
**ALL HOUSEHOLDS ARE ASKED THIS SERIES OF QS**<br><br>**b) What is the size of the pool or spa?**<br><br>▪ Large sized swimming pool (>9m x 4m wide)<br><br>▪ Medium sized swimming pool(4-9m x 2-4m wide)<br><br>▪ Small sized swimming pool (1-4m x 1-2m wide)<br><br>▪ Toddler / wading pool<br><br>• Outdoor spa<br><br>• Pond	☐<br>☐<br>☐<br>☐<br>☐<br>☐
**c) Do you top up the pool at particular times of the year? If so, for how much of the year would you top it up?**<br><br>▪ ¼ of the year<br><br>▪ 1/3 of the year<br><br>▪ ½ of the year<br><br>▪ ¾ of the year<br><br>▪ all year	☐<br>☐<br>☐<br>☐<br>☐

**d) Who in the household is usually responsible for topping up the pool?**<br><br>▪ You (Go to f)<br><br>▪ Another household member (Go to e)<br><br>▪ An employee / contractor (Go to e)	☐<br>☐<br>☐
**e) Are you able to answer questions about the number of times and length of time the pool is filled?**<br><br>▪ Yes **(Go to f)**<br><br>▪ No **(Go to CAR MODULE)**	☐<br>☐
**f) Think back over the last 7 days: did you or anyone else in the household top up the pool etc?**<br><br>▪ Yes **(Go to g)**<br><br>• No **(Go to Swimming pool Q2)**	☐<br>☐
**g) How many times did you or someone else in the household top up the pool etc in the last 7 days?**<br>**(If you answered once, Go to g, otherwise Go to h)**	#
**h) If the pool etc was topped up only once in the last week, when was the last time before this that it was topped up?**<br><br>• Between 1-2 weeks ago<br><br>• More than 2 weeks but less than 4 weeks ago<br><br>• More than a month ago	☐<br>☐<br>☐
**i) In which ways was the pool pond etc topped up in the last 7 days?**<br>▪ Hose<br><br>▪ Bucket<br><br>▪ Other Specify…………………………………	☐<br>☐<br>☐
**j) When the pool etc was last topped up in the last 7 days approximately how long was the hose running or how many buckets were used (ie per session)?**<br>**GO TO CAR MODULE**	#

**2. If you did not top up the pool etc in the last 7 days think about the occasion before that when you topped up the pool**<br><br>**a) When was this?**<br><br>• Between 1-2 weeks ago<br><br>• More than 2 weeks but less than 4 weeks ago<br><br>• More than a month ago	☐<br>☐<br>☐
**b) In which ways was the pool pond etc topped up on this occasion?**<br><br>▪ Hose<br><br>▪ Bucket<br><br>▪ Other	☐<br>☐<br>☐
**c) On this last occasion that you topped up the pool etc, approximately how long was the hose running/ how many buckets were used?**<br>**GO TO CARWASHING MODULE**	#

CAR WASHING MODULE	
**1. Do you wash cars at home using the outdoor water tap?**<br><br>▪ Yes **(if DUAL RETICULATION Go to a, If CONTROL go to b)**<br><br>▪ No **(Go to PET WASHING MODULE)**	☐<br>☐
**QUESTION FOR DUAL RETICULATION ONLY**<br>**a) Which water type do you use to clean the car?**<br><br>• **Recycled water only**<br><br>• **Drinking water only**<br><br>• **A mixture of drinking water and recycled water**	☐<br>☐<br>☐
**b) How many cars on the property?**	#
**c) How many cars are washed at home?**	#
**d) Who in the household is usually responsible for washing the car(s) at home?**<br><br>▪ You (Go to f)<br><br>▪ Another household member (Go to e)	☐<br>☐
**e) Are you able to answer questions about car washing?**<br><br>▪ **Yes (Go to f)**<br><br>▪ **No (Go to PET WASHING MODULE)**	☐<br>☐
**f) Think back over the last 7 days: did you or anyone else in the household wash the car using water from the outdoor water tap?**<br><br>▪ **Yes (Go to g)**<br><br>• **No (Go to CAR MODULE Q2)**	☐<br>☐
**g) How many times was the car washed during this 7 day period?**<br>**(if once, Go to h, otherwise Go to i)**	#

**h) If the car was washed once during the last 7 days how long ago before this was the car washed?**<br><br>▪ Between 1-2 weeks ago<br><br>▪ More than 2 weeks but less than 4 weeks ago<br><br>▪ More than a month ago	☐<br>☐<br>☐
**i) In which ways did you or someone else in the household wash the car during the last 7 days? *(More than one answer allowed)***<br>▪ Hose, no trigger nozzle<br><br>▪ Hose with trigger nozzle<br><br>▪ High pressure device<br><br>▪ Bucket	Y/N<br><br>☐<br>☐<br>☐<br>☐
**j) On this last occasion that you cleaned the car approximately how long was the hose running and / or how many buckets did you use?**<br><br>▪ Hose, no trigger nozzle<br><br>▪ Hose with trigger nozzle<br><br>▪ High pressure device<br><br>▪ Bucket	#<br><br>#<br><br>#<br><br>#
**k) What is the maximum number of people present in the vicinity when you last washed the car excluding the person that washed the car?**<br>**GO TO PET MODULE**	#
**2. If you <u>did not</u> wash the car in the last 7 days think about the occasion before that when you washed the car**<br>**a) How long ago was this?**<br><br>• Between 1-2 weeks ago<br><br>• More than 2 weeks but less than 4 weeks ago<br><br>• More than a month ago	☐<br>☐<br>☐

**b) In which of the following ways did you or someone else in the household wash the car on this occasion ?** ***(More than one answer allowed)***<br><br>▪ Hose, no trigger nozzle<br>▪ Hose with trigger nozzle<br>▪ High pressure device<br>▪ Bucket	Y/N<br><br>☐<br>☐<br>☐<br>☐
**c) On this last occasion that you cleaned the car approximately how long was the hose running and / or how many buckets did you use?**<br><br>▪ Hose, no trigger nozzle<br>▪ Hose with trigger nozzle<br>▪ High pressure device<br>▪ Bucket	#<br>#<br>#<br>#
**d) What is the maximum number of people present in the vicinity when you last washed the car excluding the person that washed the car?**<br><br>**GO TO PET MODULE**	#

PET WASHING MODULE	
**a) How many pets do you have?**	#
**b) Do you have pets that are washed or rinsed at home using the <u>outdoor water tap?</u>?**<br>▪ Yes **(Go to c)**<br>▪ No **(Go to HARD SURFACES CLEANING MODULE)**	☐<br>☐
**c) How many pets are washed or rinsed at home using the outdoor water tap?**	#
**d) What water do you use outside to wash or rinse the pet?**<br>• **Recycled water only**<br>• **Drinking water only**<br>• **A mixture of recycled and drinking water**	☐<br>☐<br>☐
**e) Who in your household is usually responsible for washing or rinsing the pet at home?**<br>▪ You (Go to g)<br>▪ Other household member (Go to f)<br>▪ Other (pet service etc) (Go to f)	☐<br>☐<br>☐
**f) Are you able to answer questions about pet washing frequency and use of water for this purpose?**<br>▪ **Yes (Go to g)**<br>▪ **No (Go to HARD SURFACES CLEANING MODULE)**	☐<br>☐
**g) Think back over the last 7 days: did you or anyone else in the household wash or rinse the pets using the outdoor water tap?**<br>▪ **Yes (Go to h)**<br>• **No (Go to hard surfaces Module Q2)**	☐<br>☐
**h) How many times in the last 7 days did you wash the pet?**<br>**(If once, Go to i otherwise Go to j)**	#

**i) If the pet was washed only once in the last 7 days when was the last time before that the pet was washed?**<br><br>▪ Between 1-2 weeks ago<br><br>▪ More than 2 weeks but less than 4 weeks ago<br><br>▪ More than a month ago	☐<br>☐<br>☐
**j) In which of the following ways did you or someone else in the household wash or rinse the pet in the last 7 days? *(More than one answer allowed)***<br><br>▪ Using bucket<br><br>▪ Using hose with trigger nozzle<br><br>▪ Using pet cleaning service that connects to outdoor recycled water supply<br><br>▪ Other<br>Specify …………………………………………	Y/N<br><br>☐<br>☐<br>☐<br>☐
**k) For how long was the water tap on when you washed the pet?**<br><br>▪ Using bucket<br><br>▪ Using hose with trigger nozzle<br><br>▪ Using pet cleaning service that connects to outdoor recycled water supply<br><br>▪ Other	#<br>#<br>#<br>#
**l) What was the maximum number of people present when you washed the pet in the last 7 days?**<br><br>**GO TO HARD SURFACES CLEANING MODULE**	#
**2. If you <u>did not</u> wash the pet in the last 7 days think about the occasion before that when you washed the pet(s)**<br><br>**a) How long ago was this?**<br><br>• Between 1-2 weeks ago<br><br>• More than 2 weeks but less than 4 weeks ago<br><br>• More than a month ago	☐<br>☐<br>☐

**b) In which of the following ways did you or someone else in the household wash or rinse the pet on this occasion? *(More than one answer allowed)***<br><br>▪ Using bucket<br><br>▪ Using hose with trigger nozzle<br><br>▪ Using pet cleaning service that connects to outdoor recycled water supply<br><br>▪ Other<br>Specify ………………………………………….	Y/N<br><br>☐<br><br>☐<br><br>☐<br><br>☐
**c) For how long was the water tap on when you washed the pet?**<br><br>▪ Using bucket<br><br>▪ Using hose with trigger nozzle<br><br>▪ Using pet cleaning service that connects to outdoor recycled water supply<br><br>▪ Other	#<br><br>#<br><br>#<br><br>#
**d) What was the maximum number of people present in the vicinity when the pet was last washed excluding the person washing the pet?**<br><br>**GO TO HARD SURFACES CLEANING MODULE**	#

HARD SURFACES CLEANING MODULE	
**1. Do you use the outdoor water tap to clean <u>outdoor</u> hard surfaces such as windows paths etc?**<br><br>▪ Yes **(Go to a if dual reticulation households or go to b if control houshold)**<br><br>▪ No **(Go to 'OTHER' MODULE)**	☐<br>☐
**ASK DUAL RETICULATION HOUSEHOLDS ONLY**<br>**a) What sort of water is used to clean outdoor hard surfaces such as windows and paths etc?**<br><br>• **Recycled water only**<br><br>• **Drinking water only**<br><br>• **A mixture of drinking water and recycled water**	☐<br>☐<br>☐
**b) Who is the person usually responsible for cleaning outdoor hard surfaces such as windows, paths etc?**<br><br>▪ You (Go to d)<br><br>• Another household member (Go to c)<br><br>▪ Contractor (Go to c)	☐<br>☐<br>☐
**c) Are you able to answer questions about the washing of outdoor hard surfaces and use of water for this purpose?**<br><br>▪ Yes **(Go to e)**<br><br>▪ No **(Go to 'Other' module)**<br><br>▪	☐<br>☐
**d) Which of the following <u>outdoor</u> hard surfaces are washed down?**<br><br>▪ Paths (concrete, brick and other)<br><br>▪ Paving surrounding swimming pools, decking, entertainment area<br><br>▪ Brick walls and masonry<br><br>▪ Windows<br><br>▪ Other<br>**Specifiy……………………………………….**	Y/N<br>☐<br>☐<br>☐<br>☐<br>☐

**e) Think back over the last 7 days: did you or anyone else in the household wash down hard surfaces outside?**<br><br>▪ **Yes (Go to f)**<br><br>▪ **No (Go to HARD SURFACES MODULE Q2)**	☐<br>☐
**f) How many times in the last 7 days did you wash down hard surfaces outside?**<br><br>**(If once, Go to f otherwise Go to g)**	#
**g) If outdoor hard surfaces were washed down only once in the last 7 days when was the last time before this that they were washed down?**<br><br>▪ Between 1-2 weeks ago<br><br>▪ More than 2 weeks but less than 4 weeks ago<br><br>▪ More than a month ago	☐<br>☐<br>☐
**h) In which of the following ways did you or someone else in the household wash down outdoor hard surfaces in the last 7 days? *(More than one answer allowed)***<br><br>▪ Hose, no trigger nozzle<br><br>▪ Hose with trigger nozzle**)**<br><br>▪ High pressure device<br><br>▪ Bucket<br><br>▪ Other<br>Specify ………………………………………….	Y/N<br>☐<br>☐<br>☐<br>☐<br>☐
**i) For how long was the water tap on (or how many buckets were used) during each session when you washed down the hard surfaces?**<br><br>▪ Hose, no trigger nozzle<br><br>▪ Hose with trigger nozzle**)**<br><br>▪ High pressure device<br><br>▪ Bucket<br><br>▪ Other	#<br>#<br>#<br>#<br>#

**j) What was the maximum number of people present in the vicinity when the hard surfaces were washed down in the last 7 days excluding the person washing them down?**<br>**GO TO 'OTHER' MODULE**	#
**2. If you or someone else <u>did not</u> wash down outdoor hard surfaces in the last 7 days think about the occasion before that when you washed hard surfaces**<br><br>**a) How long ago was this?**<br>• Between 1-2 weeks ago<br>• More than 2 weeks but less than 4 weeks ago<br>• More than a month ago	☐<br>☐<br>☐
**b) In which of the following ways did you or someone else in the household last wash down outdoor hard surfaces? *(More than one answer allowed)***<br>▪ Hose, no trigger nozzle<br>▪ Hose with trigger nozzle**)**<br>▪ High pressure device<br>▪ Bucket<br>▪ Other<br><br>Specify ………………………………………….	Y/N<br>☐<br>☐<br>☐<br>☐<br>☐
**c) For how long was the water tap on (or how many buckets were used) during each session when the hard surfaces were washed down?**<br>▪ Hose, no trigger nozzle<br>▪ Hose with trigger nozzle**)**<br>▪ High pressure device<br>▪ Bucket<br>▪ Other	#<br>#<br>#<br>#<br>#

**d) What was the maximum number of people present in the vicinity when outdoor hard surfaces were washed down excluding the person washing them down?**<br>**GO TO 'OTHER' MODULE**	#

OTHER MODULE	
**1. Do you use outdoor water taps to supply water for any purposes other than garden watering, car washing, hard surface cleaning, pet washing, such as water play?**<br><br>▪ No (**Go to Question 3 this module)**<br><br>▪ Yes **(Go to a)**	☐<br>☐
**a) Specify the activity**<br><br>………………………………	
ASK DUAL RETICULATION HOUSEHOLDS ONLY<br>**b) What type of outdoor water do you use?**<br><br>• **Recycled water only**<br><br>• **Drinking water only**<br><br>• **A mixture of recycled water and drinking water**	☐<br>☐<br>☐
**c) Who in the household conducts this activity?**<br><br>▪ You (Go to e)<br><br>▪ Other household member (Go to d)	☐<br>☐
**d) How often is this activity conducted?**<br><br>• **Weekly or more frequently**<br><br>• **Every 2-4 weeks**<br><br>• **Less frequently than monthly**	☐<br>☐<br>☐
**e) What method is used when conducting this activity? *(More than one answer allowed)***<br>▪ Hose, no trigger nozzle<br><br>▪ Hose with trigger nozzle<br><br>▪ High pressure device<br><br>▪ Bucket	Y/N<br><br>☐<br>☐<br>☐<br>☐

**f) On this last occasion that you performed this activity approximately how long was the hose running and / or how many buckets did you use?**<br>▪ Hose, no trigger nozzle<br>▪ Hose with trigger nozzle<br>▪ High pressure device<br>▪ Bucket	#<br>#<br>#<br>#
**Q3 How often do members of the family visit local parks?**<br>• **More than once per week**<br>• **Weekly (once per week)**<br>• **Every 2-4 weeks**<br>• **Less frequently than monthly**	☐<br>☐<br>☐<br>☐

LAUNDRY MODULE	
**1. Who in your household usually does the laundry tasks?**<br>▪ You (Go to question 3)<br>▪ Other householder (Go to 2)<br>▪ Other (ie no laundry facilities etc) (Go to 2)	☐<br>☐<br>☐
**2. Are you able to answer questions about laundry washing and the use of water for this purpose**<br>▪ Yes **(Go to Question 3, this section)**<br>▪ No **(Go to Question 8, this section)**	☐<br>☐
**3. What proportion of your laundry is done using a machine?**<br>▪ All or almost all<br>▪ About ¾<br>▪ About ½<br>▪ About ¼<br>▪ Almost none/ None	☐<br>☐<br>☐<br>☐<br>☐
**4. Type of washing machine?**<br>▪ Top loader<br>▪ Front loader	☐<br>☐
**5. Water level usually selected?**<br>▪ High<br>▪ Medium<br>▪ Low<br>▪ Automatic selection	Y/N<br>☐<br>☐<br>☐<br>☐
**6. Number of loads per week?**	#

**7.** **Water temperature usually selected**<br>▪ Hot only<br>▪ Cold only<br>▪ Warm	Y/N<br>☐<br>☐<br>☐
**8.** **Are there any rainwater tanks on the property?**<br>▪ Yes **(Go to Question 9, this section)**<br>▪ No **(Go to Question 10, this section)**	☐<br>☐
**9.** **What is the rainwater used for?**<br>▪ Drinking<br>▪ Lawn and garden watering<br>▪ Vegetable garden watering<br>▪ Toilet flushing<br>▪ Other …………………………….(specify)<br>STOP HERE FOR RECYCLED WATER HOUSEHOLDS, CONTINUE FOR CONTROL HOUSEHOLDS	Y/N<br>☐<br>☐<br>☐<br>☐<br>☐
**10.** **Do you have a grey water or stormwater system**<br>• **Yes (Go to QUESTION 11)**<br>• **No (FINISH)**	☐<br>☐
**11.** **What is the grey water or stormwater used for?**<br>▪ Lawn and garden watering<br>▪ Vegetable garden watering<br>▪ Toilet flushing<br>Other (specify)…………………………………	Y/N<br>☐<br>☐<br>☐<br>☐

End of the interview

This is the end of the interview but I would like to explore whether you are willing to complete a water activity diary for a one week period in the coming month.

If No response,

Thank householder for their time in assisting with the telephone interview and ask if there are any questions

If Yes response:

Confirm the residential address to which diary cards should be sent
Agree on week in the coming month that the diary will be completed

Advise householder of $40 department voucher for return of a **satisfactorily completed** diary. Emphasise that ***all*** water usage must be recorded for the week and that **all** boxes must be filled in. If there is no water usage for the week for a particular purpose eg outdoor purposes other than garden watering or garden watering then instruct householder to indicate this by placing a diagonal line on the card.
