# Supplementary material for: Collecting household water usage data: telephone questionnaire or diary?
Source: BMC Med Res Methodol. 2009 Nov 9;9:72. doi: 10.1186/1471-2288-9-72 (PMC2777918; doi:10.1186/1471-2288-9-72)
Supplement: Additional file 2 — Diary cards. This file contains the diary cards (five different cards) sent to householders that comprised the water-activity diary. [file 1471-2288-9-72-S2.pdf]

HOUSEHOLDER NUMBER DIARY CARD

HOUSEHOLD CODE NO:
0001

Day, date and time <u>all</u> diary cards commenced:
Wednesday 17/7/05 7.00am

<u>Please enter a number into each box even if zero</u>

Day	Numbers of people overnight	
	18 years old and older	17 years and younger
**Monday**	2	0
**Tuesday**	2	0
**Wednesday**	2	0
**Thursday**	3	0
**Friday**	2	0
**Saturday**	2	2
**Sunday**	2	0

Sample

Number of recycled water taps	Outside house	Inside house
	**2**	**0**

HOUSEHOLDER NUMBER DIARY CARD

HOUSEHOLD CODE NO:

Day, date and time <u>all</u> diary cards commenced:
…………………………………………………………………

<u>Please enter a number into each box even if zero</u>

Day	Numbers of people overnight	
	18 years old and older	17 years and younger
**Monday**		
**Tuesday**		
**Wednesday**		
**Thursday**		
**Friday**		
**Saturday**		
**Sunday**		

Number of recycled water taps	Outside house	Inside house

HOUSEHOLD CODE NO: 0001

LAUNDRY (WASHING MACHINE) DIARY CARD

Day, date and time diary commenced: Wednesday 17/7/05 7.00am

Please enter a tick against the appropriate day each time your clothes are washed

**Machine type (circle)**	Front loader (circled)	Top loader
**Brand and Model**	Asko HY123	

Sample

**Day**	**Water level**				**Water temperature**			**Cycle type**	
**Code**	**L**	**M**	**H**	**A**	**C**	**W**	**H**	**DD**	**SD**
**Monday**	√					√		√	
			√				√		√
**Tuesday**									
**Wednesday**			√				√		√
**Thursday**			√				√		√
			√				√		√
			√√				√√		√√
**Friday**									
**Saturday**	√					√		√	
**Sunday**	√					√		√	

CODES TO USE

**Water level**	**Water Temperature**	**Cycle types**
L = Low	C = Cold	DD = drip dry
M = Medium	W = Warm	SD= spin dry
H = High	H = Hot	
A = Automatic		

Where Nº of loads is more than 3 a day enter additional ticks in box see Thursday sample

HOUSEHOLD CODE NO:

LAUNDRY (WASHING MACHINE) DIARY CARD

Day, date and time diary commenced:……………………………

Please enter a tick against the appropriate day each time your clothes are washed

**Machine type (circle)**	Front loader	Top loader
**Brand and Model**		

**Day**	**Water level**				**Water temperature**			**Cycle type**	
**Code**	**L**	**M**	**H**	**A**	**C**	**W**	**H**	**DD**	**SD**
**Monday**									
**Tuesday**									
**Wednesday**									
**Thursday**									
**Friday**									
**Saturday**									
**Sunday**									

CODES TO USE

**Water level**	**Water Temperature**	**Cycle types**
L = Low	C = Cold	DD = drip dry
M = Medium	W = Warm	SD= spin dry
H = High	H = Hot	
A = Automatic		

Where Nº of loads is more than 3 a day enter additional ticks in box see Thursday sample

OUTDOOR GARDEN WATERING DIARY CARD

HOUSEHOLD CODE NO
0001

Day, date and time diary card commenced: Wednesday 17/7/05 7.00am

Water used for garden watering (tick one box):

Recycled water only [√] **Drinking water only** [ ] **Both recycled and drinking water** [ ]

	Monday			Tuesday			Wednesday			Thursday			Friday			Saturday			Sunday		
**Method**	**AS**			**AS**	**WCB**		**AS**			**AS**	**HH**		**AS**	**HH**		**AS**			**AS**		
**Duration (minutes or number of buckets)**	**20m**			**20m**	**10buckets**		**20m**			**20m**	**10m**		**20m**	**10m**		**20m**			**20m**		
**Total number people in garden**	**0**			**0**	**1**		**0**			**0**	**2**		**0**	**2**		**0**			**0**		

Sample

METHOD CODES TO USE:

**AS** = Automatic box irrigation controller	**MS** = Manual operation, tap timer
**HH** = Hand held hose (with or without nozzle)	**HS** = Hose and sprinkler
**ES** = Electronic tap timer	**WCB** = Watering can or bucket
**SH =** Soaker Hose	

OUTDOOR GARDEN WATERING DIARY CARD

HOUSEHOLD CODE NO

Day, date and time diary card commenced:………………………………

Water used for garden watering (tick one box):

Recycled water only [ ] **Drinking water only** [ ] **Both recycled and drinking water** [ ]

	Monday			Tuesday			Wednesday			Thursday			Friday			Saturday			Sunday		
**Method**																					
**Duration (minutes or number of buckets)**																					
**Total number people in garden**																					

METHOD CODES TO USE:

**AS** = Automatic box irrigation controller	**MS** = Manual operation, tap timer
**HH** = Hand held hose (with or without nozzle)	**HS** = Hose and sprinkler
**ES** = Electronic tap timer	**WCB** = Watering can or bucket
**SH =** Soaker Hose	

OUTDOOR USAGE DIARY CARD

HOUSEHOLD CODE NO
0001

Day, date and time diary card commenced: Wednesday 17/7/05 7.00am

Type of water used for outdoor purposes detailed below (tick one box):

Recycled water only [√] **Drinking water only** [ ] **Both recycled and drinking water** [ ]

Sample

Activity		Monday	Tuesday	Wednesday	Thursday	Friday	Saturday	Sunday
**CAR WASH**	Method				**HH**			
	Duration				**10min**			
	Nº persons				**2**			
**HARD SURFACE WASH (eg. Paths, windows)**	Method				**HPD**			
	Duration				**20min**			
	Nº persons				**2**			
**OTHER Specify Pet washing**	Method					**WCB**		
	Duration					**9buckets**		
	Nº persons					**2**		
**OTHER Specify Pond fill**	Method		**HH**					
	Duration		**20min**					
	Nº persons		**2**					

CODES TO USE:

**Method**	**HH = Hand held hose (with or without nozzle)**	**WCB = Watering can or bucket**	**HPD= High pressure device**
**Other**	**Swimming pool, pond, fountain fill, pet water, boat wash, pet wash / rinse etc**	**Duration: In minutes or total number of buckets used**	**Nº persons: Number of persons present for all or part of the activity**

To fill in card see sample diary card on reverse

OUTDOOR USAGE DIARY CARD

HOUSEHOLD CODE NO

Day, date and time diary card commenced: ……………………………………

Type of water used for outdoor purposes detailed below (tick one box):

Recycled water only [ ] **Drinking water only** [ ] **Both recycled and drinking water** [ ]

Activity		Monday	Tuesday	Wednesday	Thursday	Friday	Saturday	Sunday
**CAR WASH**	Method							
	Duration							
	Nº persons							
**HARD SURFACE WASH (eg. Paths, windows)**	Method							
	Duration							
	Nº persons							
**OTHER Specify ……………….**	Method							
	Duration							
	Nº persons							
**OTHER Specify ………………**	Method							
	Duration							
	Nº persons							

CODES TO USE:

**Method**	**HH = Hand held hose (with or without nozzle)**	**WCB = Watering can or bucket**	**HPD= High pressure device**
**Other**	**Swimming pool, pond, fountain fill, pet water, boat wash, pet wash / rinse etc**	**Duration: In minutes or total number of buckets used**	**Nº persons: Number of persons present for all or part of the activity**

TOILET DIARY CARD

Toilet location: Ensuite bathroom
Separate room:……....~~Yes~~/No

HOUSEHOLD CODE NO
0001

Day, date and time commenced Wednesday 17/7/05 7.00am

- **Please enter a tick against the appropriate day each time the toilet is flushed either against full or half flush**

sample

Day		1	2	3	4	5	6	7	8	9	10	11	12	13	14	15	16	17	18	19	20	21	22
**Monday**	Full	√	√	√	√	√																	
	Half	√	√	√	√	√	√	√	√	√	√												
**Tuesday**	Full	√	√	√																			
	Half	√	√	√	√	√	√	√	√	√	√												
**Wednesday**	Full	√	√	√	√																		
	Half	√	√	√	√	√	√	√	√	√	√												
**Thursday**	Full	√	√	√	√																		
	Half	√	√	√																			
**Friday**	Full	√	√	√	√	√	√																
	Half	√	√	√	√	√	√	√															
**Saturday**	Full	√	√	√	√	√	√	√	√	√	√												
	Half	√	√	√	√	√	√	√	√	√	√	√	√	√	√	√	√						
**Sunday**	Full	√	√	√	√	√	√	√	√	√	√	√	√	√									
	Half	√	√	√	√	√	√	√	√	√	√	√	√	√	√	√	√						

TOILET DIARY CARD

Toilet location:..………………………………..
Separate room: ……....**Yes /No**

HOUSEHOLD CODE NO

Day, date and time commenced:………………………………

- **Please enter a tick against the appropriate day each time the toilet is flushed either against full or half flush (See sample diary card on reverse)**

Day		1	2	3	4	5	6	7	8	9	10	11	12	13	14	15	16	17	18	19	20	21	22
**Monday**	Full																						
	Half																						
**Tuesday**	Full																						
	Half																						
**Wednesday**	Full																						
	Half																						
**Thursday**	Full																						
	Half																						
**Friday**	Full																						
	Half																						
**Saturday**	Full																						
	Half																						
**Sunday**	Full																						
	Half																						
